# Supplementary material for: A set of multi-entry identification keys to African frugivorous flies (Diptera, Tephritidae)
Source: Zookeys. 2014 Jul 24;(428):97–108. doi: 10.3897/zookeys.428.7366 (PMC4143993; doi:10.3897/zookeys.428.7366)
Supplement: Supplementary material 6 — Key to Ceratitis [file zookeys-428-097-s006.zip › SF6_ZooKeys_key to Ceratitis/key/SF6_ZooKeys_key to Ceratitis/Media/Html/Ceratitis cornuta.htm]

Ceratitis (Ceratitis) cornuta (Bezzi)


***Ceratitis*** **(*Ceratitis*) *cornuta*
(Bezzi)**

*Pterandrus cornutus* Bezzi, 1924: 478.

Body
length: 4.33 (4.00-4.65) mm; wing length: 4.46 (4.20-4.70) mm.

Male

Head.
Antenna yellow. Third antennal segment twice as long as second segment. Arista
with short hairs, slightly longer than in other *Ceratitis*. Frons pale
yellow; with short scattered hairs which are largely the same colour as frons.
Frontal, ocellar and orbital bristles yellowish red, upper orbital sometimes
dark; lower orbital with variable tip shape: pointed, or flattened; frontal
bristles weakly developed. Face pale yellow. Genal bristle pale; genal setulae
pale, weakly developed. Postocellar and outer vertical bristles pale.

Thorax.
Postpronotum white, with distinct black spot. Mesonotum: ground colour orange,
occasionally more orange-brown, microtrichiae pattern silvery with orange
shine, mesonotal spots more brownish; sutural yellow spots occasionally
extended as postsutural lines, two separate prescutellar yellow lunules.
Scapular setae pale. Scutellum yellow-white, basally with two dark separate
spots, apically with three merged spots, incisions deep, almost reaching apical
bristles. Anepisternum with lower half darker yellow but not distinct, pilosity
in lower half darker yellow. 

Legs
yellow; setation typical for subgenus, mainly pale especially on femora. Fore
femur posteriorly with weakly developed bush of longer orange-coloured hairs
along entire length, posterodorsal hairs longer; ventral spines yellow-orange;
anteroventrally without row of hairs. Mid femur with few longer hairs basally
along ventral side but no distinct row. Hind femur with longer hairs dorsally
and ventrally on apical fourth. 

Wing,
brownish marking slightly more reduced than in other *Ceratitis* species.
Banding, marginal continuous or with partial interruption (rarely completely
interrupted); cubital and medial bands free; crossvein r-m at middle of discal
cell. Vein R1 beyond or equal with crossvein r-m. Crossvein dm-cu posteriorly
more outwards than anteriorly.

Abdomen.
Yellow. Setation and banding typical for subgenus.

Female

As
in male except for the following characters: Frons convex to flat, slightly
darker than in male. Frontal, ocellar and orbital bristles all dark; orbitals
not modified, frontal bristles well developed. Genal bristles pale or dark.
Postocellar and outer vertical bristles dark. Genal bristle dark or pale; genal
setulae dark, strongly developed. Outer scapular black. Incisions apical
scutellar spots variable but not as deep as in male; basal spots narrowly
touching. Legs without feathering. Oviscape shorter than abdominal terga. 

(Description
after De Meyer, 2000)
